# Low Electric Treatment activates Rho GTPase via Heat Shock Protein 90 and Protein Kinase C for Intracellular Delivery of siRNA

**DOI:** 10.1038/s41598-019-40904-z

**Published:** 2019-03-11

**Authors:** Mahadi Hasan, Susumu Hama, Kentaro Kogure

**Affiliations:** 1 Department of Biophysical Chemistry, Kyoto Pharmaceutical University, Kyoto, 607-8414 Japan; 2 Department of Pharmaceutical Health Chemistry, Tokushima University Graduate School of Biomedical Sciences, Tokushima, 770-8505 Japan; 3 JSPS International Research Fellow, Tokushima, Japan

## Abstract

Low electric treatment (LET) promotes intracellular delivery of naked siRNA by altering cellular physiology. However, which signaling molecules and cellular events contribute to LET-mediated siRNA uptake are unclear. Here, we used isobaric tags in relative and absolute quantification (iTRAQ) proteomic analysis to identify changes in the levels of phosphorylated proteins that occur during cellular uptake of siRNA promoted by LET. iTRAQ analysis revealed that heat shock protein 90 (Hsp90)α and myristoylated alanine-rich C-kinase substrate (Marcks) were highly phosphorylated following LET of NIH 3T3 cells, but not untreated cells. Furthermore, the levels of phosphorylated Hsp90α and protein kinase C (PKC)γ were increased by LET both with siRNA and liposomes having various physicochemical properties used as model macromolecules, suggesting that PKCγ activated partly by Ca^2+^ influx as well as Hsp90 chaperone function were involved in LET-mediated cellular siRNA uptake. Furthermore, LET with siRNA induced activation of Rho GTPase via Hsp90 and PKC, which could contribute to cellular siRNA uptake accompanied by actin cytoskeleton remodeling. Collectively, our results suggested that LET-induced Rho GTPase activation via Hsp90 and PKC would participate in actin-dependent cellular uptake of siRNA.

## Introduction

Functional nucleic acids such as a short interfering RNA (siRNA) could have applications as next-generation drugs to treat various diseases^1,2^. For successful clinical application of therapeutic siRNA, effective methods for delivery of exogenous nucleic acids to target cells are needed^1^. The intracellular delivery efficiencies of naked siRNA are extremely low due to the negative charge of these macromolecules^3,4^. Thus, to achieve efficient delivery of siRNA into target cells, many carrier- or physical stimulus-based methods have been developed^1,5–9^. Liposomes and micelles are representative siRNA carriers that promote delivery of siRNA into cells through a positive surface charge that facilitates electrostatic interactions with cellular membranes. Such lipid-based carriers can also control intracellular trafficking of siRNA to various cellular compartments and organelles, including mitochondria^10^, although the cationic materials comprising these carriers can induce cytotoxicity and a non-specific RNA interference (RNAi) effect^11,12^.

To overcome these undesired side effects, various physical stimuli that can promote siRNA delivery without use of carriers have been explored^8,13,14^. We previously reported that delivery of naked siRNA mediated by treatment with low electricity (LET), known as iontophoresis, induces an RNAi effect in the skin of animal models through the inhibition of expression of targeting genes^15^. The low density of the electric current (0.5 mA/cm^2^ or less) used in LET-mediated delivery of naked siRNA is lower than that for conventional electroporation. Although electroporation can introduce various drugs and nucleic acids into cells via the formation of microscopic pore in plasma membrane by applying high voltage pulse (≥100 V)^16^, the membrane damage leading to cytotoxicity hampers the clinical application. To recover such membrane damage, it takes at least 72 h even in healthy cells that are relatively low sensitivity to electroporation-induced cytotoxicity^17^. Therefore, the precise optimization of protocol is needed for the drugs and nucleic acids delivery using electroporation. Wang, R. J. *et al*. reported that the membrane penetration of indomethacin mediated by LET was more potent than that by electroporation^18^. Although cellular uptake efficacy of nucleic acids between LET and electroporation is needed to be directly compared, LET without cytotoxicity is expected to be more useful drug delivery methods than electroporation. Alternatively LET can nonetheless deliver naked siRNA into targeted cells without the cytotoxicity that can accompany electroporation^19^. Moreover, we previously reported that various functional nucleic acids including siRNA delivered by LET have their functionality in the cells^19,20^. However, the detailed mechanism involved in cellular uptake of siRNA following LET remains unclear. The cellular uptake of naked siRNA promoted by LET is accompanied by changes in membrane potential mediated by transient receptor potential (TRP) channels^19^ that facilitate cellular influx of cationic ions such as calcium ions in response to extracellular stimuli^21^. We previously showed that protein kinase C (PKC) is activated through influx of calcium ions into cells during transdermal delivery of cationic liposomes by LET^22^. PKC-associated signal transduction contributes to cellular uptake of macromolecules via actin remodeling^23–25^ and calcium ions act as second messengers that meditate various signal transduction and cellular events for cellular uptake of macromolecules^26,27^. Thus, LET could induce changes in cellular physiology that result in activation of various signal transduction pathways. Furthermore, changes in the phosphorylation state of proteins involved in such pathways could affect the efficiency of cellular siRNA uptake.

To clarify the cellular uptake mechanisms involved in LET, in the current study we used isobaric tags for relative and absolute quantification (iTRAQ) proteomic analysis to identify proteins that have altered phosphorylation status following LET. iTRAQ is a comprehensive protein analysis technique that involves binding of up to eight types of isobaric tags to peptides and subsequent MS/MS analysis to allow quantitative comparisons of the identified proteins^28^. Similar approaches are widely used in a variety of settings, including identification of serum biomarkers for lung cancer, metabolic analyses in heart mitochondria and signaling pathway analyses in the endoplasmic reticulum^29–31^. In this study we also show that the proteins showing up-regulated phosphorylation following LET promote actin cytoskeleton remodeling via activation of Rho GTPase that is associated with cellular siRNA uptake.

## Results

### iTRAQ analysis of phosphorylated proteins upregulated by LET

**Figure 1:**
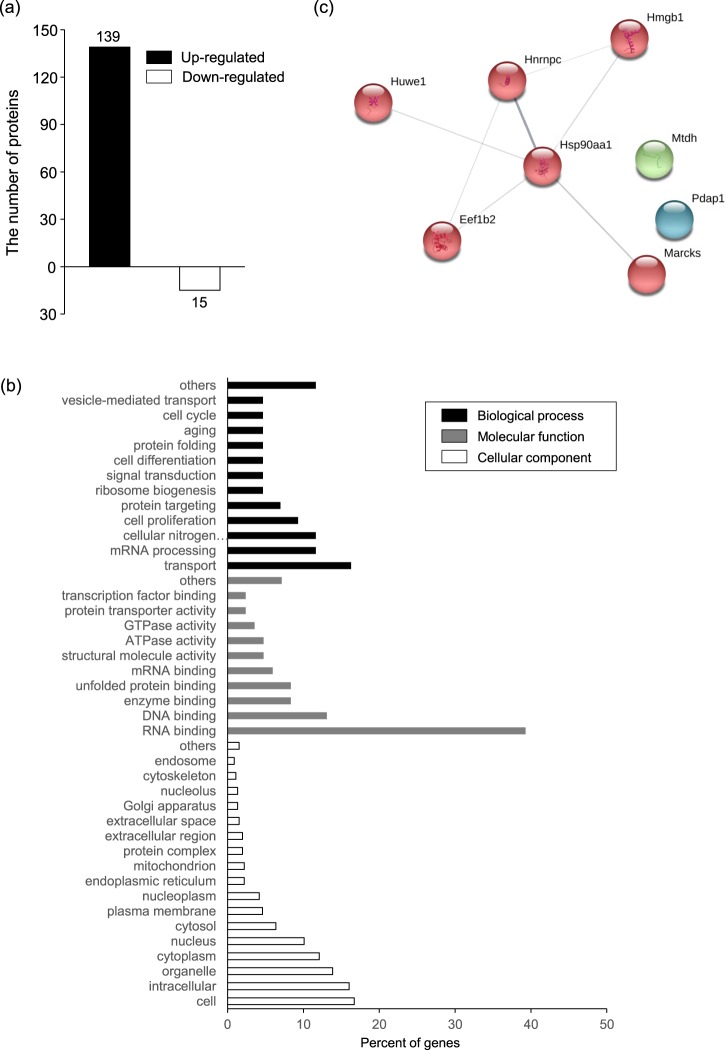
Characteristics of phosphorylated proteins induced by LET. Protein samples for iTRAQ analysis were extracted from non- and LET-treated cells. Proteins in LET-exposed cells having phosphorylation levels that were increased or decreased by at least 1.2-fold relative to untreated cells and >0.62 in Unused Prot Score were classified as having up- or down-regulation of phosphorylation. (**a**) The number of up- or down-regulated phosphoproteins in LET samples compared with untreated samples. (**b**) GO slim analysis classification of 139 up-regulated phosphoproteins by biological process, molecular function and cellular component. (**c**) STRING analysis of GO term-attached proteins among the 15 phosphoproteins showing the largest amount of upregulation following LET.

To identify proteins with altered phosphorylation status following LET, we performed an iTRAQ analysis of NIH 3T3 cells treated with LET. In this study, thresholds for detecting proteins with altered phosphorylation levels following LET were: 1.2 fold-change in levels of phosphorylated protein levels in LET-treated cells compared to untreated cells, and Unused Prot Score >0.62 that is indicative of the reliability of the identified proteins. Here, the number of proteins showing upregulated and downregulated phosphorylation following LET was 139 and 15, respectively (Fig. 1a). We next classified the proteins showing upregulated phosphorylation using a GO slim analysis to understand the biological function of the proteins (Fig. 1b). We previously reported that LET promotes cellular uptake of siRNA via an energy-dependent endocytic pathway^19^. One protein associated with vesicle-mediated transport in biological processes is Ras-related protein Rab-10 (Rab10) that has GTPase activity and is a key regulator of intracellular membrane trafficking^32,33^. Here we found that the level of phosphorylated Rab10 was 1.312-fold higher in cells exposed to LET relative to untreated cells. The 15 proteins that had the largest upregulation in phosphorylation following LET were: filamin C (Flnc), isoform 5 of heterogeneous nuclear ribonucleoproteins C1/C2 (Hnrnpc), protein Njmu-R(5730455P16Rik), 28 kDa heat- and acid-stable phosphoprotein (Pdap1), protein LYRIC (Mtdh), high mobility group protein B1 (Hmgb1), myristoylated alanine-rich C-kinase substrate (Marcks), elongation factor 1-β (Eef1b2), AHNAK nucleoprotein isoform 1 (Ahnak), heat shock protein HSP 90-α (Hsp90aa1 or Hsp90α), stress-70 protein, mitochondrial (Hspa9), poliovirus receptor (Pvr), Isoform 1 of E3 ubiquitin-protein ligase HUWE1 (Huwe1), protein AHNAK2 (Ahnak2) and pumilio 2 (Pum2) (Table 1). Given the challenges in using GO slim analysis of GO term-attached proteins to identify proteins that contribute to intracellular delivery of macromolecules (Supplemental Table S1), we used STRING analysis of the top 15 phosphorylated proteins to characterize interactions between them. Hsp90aa1 (Hsp90α) interacted with the highest number of proteins, including Hnrnpc, Hmgb1, Marcks, Eef1b2 and Huwe1 (Fig. 1c). Although previous reports suggested that Hsp90 is involved in vesicular transport^34^, whether LET-induced Hsp90 phosphorylation contributes to cellular uptake of siRNA was unclear.

**Table 1:** Proteins showing largest upregulation of phosphorylation following LET.

UniProt ID	Name	Fold-change (vs. non-treatment)
OTTMUSP00000025498	Flnc: Filamin C, γ, Uncharacterized protein	4.148
Q9Z204	Hnrnpc: Isoform 5 of Heterogeneous nuclear ribonucleoproteins C1/C2	2.849
Q9CYI0	5730455P16Rik: Protein Njmu-R	2.811
Q3UHX2	Pdap1: 28 kDa heat- and acid-stable phosphoprotein	2.470
Q80WJ7	Mtdh: Protein LYRIC	2.423
P63158	Hmgb1: High mobility group protein B1	2.279
P26645	Marcks: Myristoylated alanine-rich C-kinase substrate	2.271
O70251	Eef1b2: Elongation factor 1-β	2.192
NP_033773	Ahnak: AHNAK nucleoprotein isoform 1	2.126
P07901	Hsp90aa1(Hsp90α): Heat shock protein HSP 90-α	2.064
NP_034611	Hspa9: stress-70 protein, mitochondrial	2.032
OTTMUSP00000041471	Pvr: poliovirus receptor	2.032
Q7TMY8	Huwe1: Isoform 1 of E3 ubiquitin-protein ligase HUWE1	1.992
XP_003085889	Ahnak2: protein AHNAK2	1.927
OTTMUSP00000049811	Pum2: Pumilio 2, Uncharacterized protein	1.886

### Cellular uptake of siRNA and various macromolecules via Hsp90α activation induced by LET

**Figure 2:**
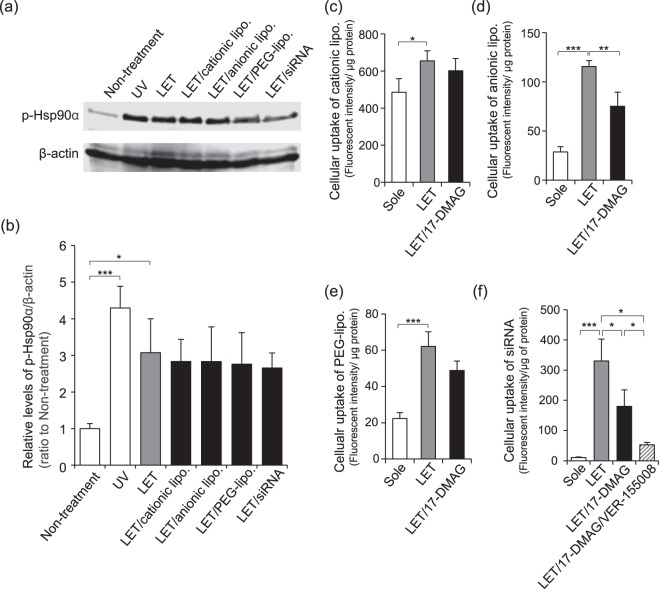
Activation of Hsp90α and cellular uptake of macromolecules by LET. (**a**,**b**) NIH 3T3 cells were treated with LET in presence of cationic lipo, anionic lipo, PEG-lipo or siRNA. Hsp90α (Thr^5/7^) levels were determined by western blotting. β-actin was used as a loading control. Samples from UV-exposed cells were used as a positive control. (**a**) Typical images from at least 3 individual experiments. Full-length blots/gels are shown in Supplementary Fig. S1. (**b**) Relative levels of p-Hsp90α/β-actin. Data are shown as the ratio to untreated cells. (**c**–**e**) Cells were pretreated for 1 h with 10 μM 17-DMAG prior to LET in the presence of cationic lipo (**c**), anionic lipo (**d**), or PEG-lipo (**e**). (**f**) Cells were pretreated with 10 μM 17-DMAG or 10 μM 17-DMAG with 50 μM VER-155008 for 1 h prior to LET in the presence of siRNA. (**c**–**f**) After incubation for 45 min, the cells were lysed, and the fluorescence intensity of the lysate was measured to evaluate cellular uptake of macromolecules. Data are shown as the fluorescence intensity divided by protein amount. Values represent the means of 3 individual experiments. Bars represent standard deviations. *P < 0.05, **P < 0.01 and ***P < 0.001.

We next examined how changes in Hsp90α phosphorylation status affected uptake of siRNA and macromolecules having different physicochemical properties such as liposomes. iTRAQ analysis revealed that Hsp90α phosphorylation was upregulated only in cells exposed to LET and not untreated cells (Table 1). Western blotting with UV-treated cells as a positive control for Hsp90α phosphorylation showed that the level of phosphorylated Hsp90α (Thr^5/7^) increased by over 2-fold for both LET with and without siRNA relative to untreated control cells (Fig. 2a,b), suggesting that the presence of siRNA did not influence Hsp90α phosphorylation induced by LET. siRNA is a negatively charged, hydrophilic macromolecule^3^. To further understand how the surface charge and hydration of macromolecules affected LET-induced Hsp90α phosphorylation, cells were exposed to LET in the presence of cationic liposomes (cationic-lipo) and anionic liposomes (anionic-lipo) as charged macromolecules, and polyethylene glycol-modified liposomes (PEG-lipo) that have a hydrated surface layer. The levels of phosphorylated Hsp90α seen following LET in the presence of various macromolecules were similar to those obtained for LET both with and without siRNA (Fig. 2a,b), suggesting that the physicochemical properties of siRNA did not affect LET-induced Hsp90α phosphorylation.

To determine whether Hsp90α participates in LET-mediated cellular uptake of siRNA, we investigated the effect of the Hsp90 inhibitor 17-DMAG on cellular uptake of various macromolecules. The cellular uptake of various macromolecules was significantly enhanced by LET (Fig. 2c–f), and the cellular uptake of siRNA following LET was over 30 times higher than that of cells treated with siRNA alone (Fig. 2f), which was the highest seen among the macromolecules examined in this study. Furthermore, co-treatment with 17-DMAG inhibited LET-mediated cellular uptake of siRNA as well as anionic-lipo (Fig. 2d,f). These results suggested that Hsp90 activation contributed to LET-mediated cellular uptake of siRNA as well as anionic-lipo.

To further understand the siRNA cellular uptake mechanism via Hsp90 activation, we examined the effect of co-treatment of Hsp90 inhibitor 17-DMAG and Hsp70 inhibitor VER-155008 on the cellular uptake of siRNA. It is known that the inactivated Hsp90 is recovered by Hsp70^35^. If the inhibitory effect of 17-DMAG on the LET-mediated cellular uptake of will be attenuated by Hsp70, co-treatment of Hsp70 inhibitor VER-155008 with 17-DMAG will be more effective. As shown in Fig. 2f, co-treatment of VER-155008 with 17-DMAG prevented LET-mediated cellular uptake by over 80%, and the inhibitory effect was more potent that treatment of only 17-DMAG. This result suggested that Hsp90 activation was involved in LET-mediated cellular uptake of siRNA.

### Involvement of PKC phosphorylation following LET-mediated Ca^2+^ influx in cellular uptake of siRNA

**Figure 3:**
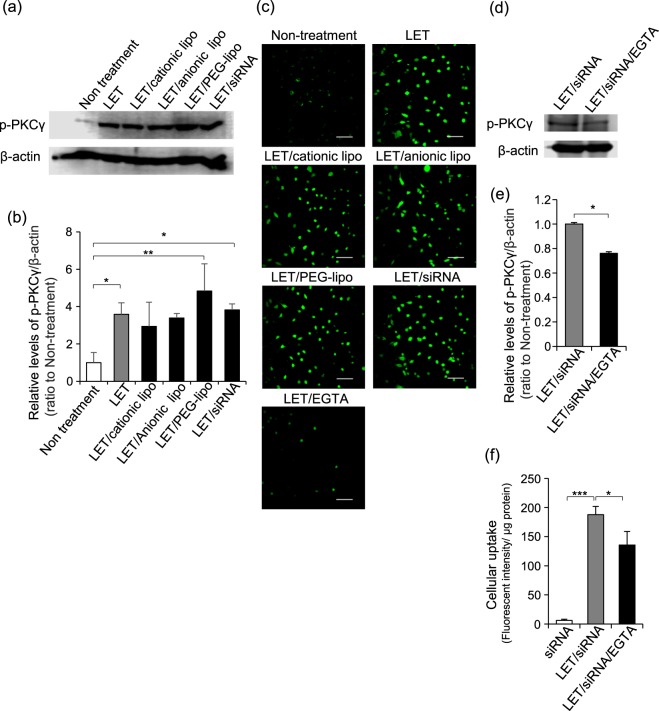
Phosphorylation of PKCγ and intracellular amount of Ca^2+^ after LET with or without macromolecules. (**a**,**b**,**d**,**e**) Levels of phosphorylated PKCγ were determined by western blotting with β-actin as a loading control. (**a**,**d**) Typical images from at least 3 individual experiments. Full-length blots/gels are shown in Supplementary Figs S2 and S3. (**b**,**e**) Relative levels of p-PKCγ/β-actin. Data are shown as the ratio to untreated cells. (**c**) Ca^2+^ influx was visualized by staining with fluo-4 fluorescent dye immediately after LET with or without macromolecules. Green signals show intracellular Ca^2+^. Scale bar indicates 100 μm. (**f**) Effect of EGTA on LET-mediated cellular uptake of siRNA. Cells were pretreated with EGTA for 30 min prior to LET in presence of rhodamine-labeled siRNA. After incubation for 45 min, cells were lysed, and fluorescence intensity in the lysate was measured. Values represent the means of 3 individual experiments. Bars represent standard deviations. *P < 0.05, **P < 0.01 and ***P < 0.001.

In the iTRAQ analysis conducted here, levels of phosphorylated Marcks, which is a substrate for many PKC isoforms^36^, increased by over 2-fold in cells exposed to LET relative to untreated cells (Table 1). This finding suggests that PKC could also be a candidate signaling molecule that is affected by LET-mediated cellular uptake of macromolecules such as siRNA. Here, we first investigated whether PKCγ is phosphorylated by LET in the presence and absence of various macromolecules. Western blotting analysis with anti-phospho PKCγ (Thr^514^) antibody indicated that the level of phosphorylated PKCγ increased by 3-fold following LET, and these levels were not affected by the presence of siRNA or other macromolecules (Fig. 3a,b). Next, to clarify the mechanism of PKCγ phosphorylation, we used fluo-4 fluorescent dye to examine Ca^2+^ influx into the cells immediately after LET with or without various macromolecules. The fluorescent signals were equivalent to the amount of intracellular Ca^2+^ induced by LET either with or without various macromolecules, whereas the enhanced fluorescent signals following LET alone were abolished by pretreatment with the Ca^2+^ chelator EGTA (Fig. 3c). Increases in PKCγ phosphorylation following LET in the presence of siRNA were prevented by EGTA pretreatment (Fig. 3d,e), suggesting that Ca^2+^ inflow contributed to the PKCγ phosphorylation induced by LET with siRNA. Moreover, EGTA pretreatment also inhibited LET-dependent enhancement in cellular uptake of siRNA (Fig. 3f). Together these results suggested that LET induced Ca^2+^ influx into cells regardless of the presence of macromolecules having different physicochemical properties and that LET enhanced the cellular uptake of siRNA through PKCγ activation induced by Ca^2+^ influx. However, the phosphorylation of PKCγ induced by LET/siRNA was not completely inhibited by blocking Ca^2+^ inflow, while the statistical significance was observed. Moreover, the cellular uptake of siRNA was not effectively inhibited by the treatment of EGTA, being correlated with the insufficient inhibition of PKCγ phosphorylation (Fig. 3f). It is known that PKCγ is phosphorylated by diacylglycerol as well as Ca^2+^ ^37^. To clarify whether PKCγ is activated by except Ca^2+^ inflow in the cells treated with LET/siRNA, we examined the effect of other PKC inhibitor Gö6983 that competitively inhibits ATP binding to the catalytic domain of PKC^37^. As shown in Supplementary Fig. S5, LET-mediated cellular uptake of siRNA was more potently inhibited by the treatment of Gö6983 in comparison with EGTA, suggesting that LET-induced Ca^2+^ inflow-mediated is partly involved in the cellular uptake of siRNA via PKCγ activation.

Next, we examined the effect of Ca^2+^ influx-mediated PKCγ activation on Hsp90α phosphorylation in cells exposed to LET in the presence of siRNA. As mentioned above, STRING analysis indicated that Hsp90α interacts with the PKC substrate Marcks, but whether Hsp90α and PKCγ interact was unclear. The level of phosphorylated Hsp90α in cells exposed to LET and siRNA was not affected by pretreatment with either EGTA or the PKC inhibitor chelerythrine, suggesting that Hsp90α phosphorylation associated with LET involves a pathway that is independent of PKCγ activated by Ca^2+^ inflow (Supplementary Fig. 4a,b). These results indicate that LET-mediated cellular uptake of siRNA could be differentially enhanced by Hsp90α and PKCγ activation.

In addition to NIH 3T3 cells, the LET-mediated cellular uptake of siRNA in mouse melanoma cell line B16-F1 was also inhibited by the treatment of Hsp90 inhibitor, calcium chelator or PKC inhibitor (Supplementary Fig. 6). It is suggested that the involvement of these signaling molecules in LET-mediated cellular uptake is not specific to NIH3T3 cells, although further investigations are needed.

### Effect of LET with siRNA on actin cytoskeleton remodeling

**Figure 4:**
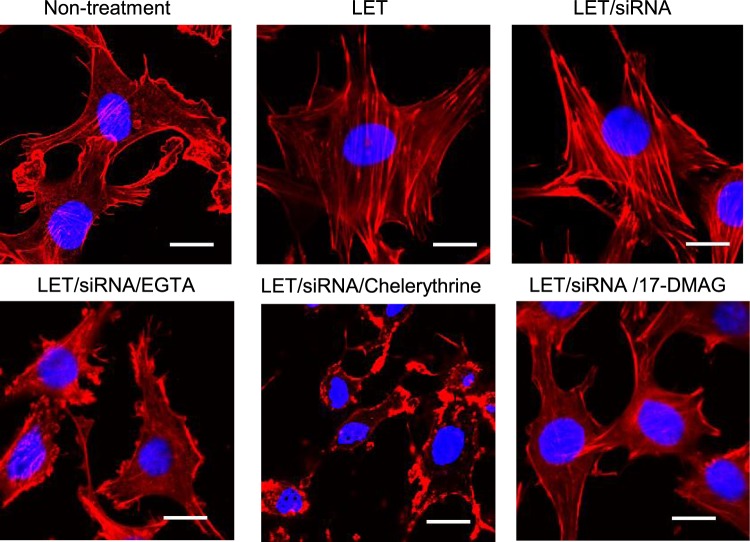
Actin cytoskeleton remodeling by LET with siRNA. Cells were treated with LET in the presence of siRNA, followed by actin staining. Actin cytoskeleton remodeling was evaluated by CLSM observation. Red and blue signals indicate rhodamine phalloidin-labelled actin and nuclei, respectively. Scale bars indicate 20 μm.

To understand cellular events activated by Hsp90α or PKCγ in LET-mediated cellular uptake of siRNA, we focused on the actin cytoskeleton that is linked to the plasma membrane^38^. Actin cytoskeleton remodeling is an essential step in endocytosis and is activated by various signaling molecules, including Hsp90 and PKC^23,39^. Staining of cells exposed to LET in the presence of siRNA with rhodamine-labeled phalloidin, which binds F-actin, yielded fluorescent signals from fiber-like structures observed at the neighboring plasma membranes as well as in the cytoplasm, whereas for cells exposed to LET alone, the fluorescent signals were limited to the cytoplasm (Fig. 4). These results indicated that LET with siRNA induced actin cytoskeleton remodeling that involved formation of lamellipodia and stress fibers associated with polymerization of cellular actin. The actin cytoskeleton remodeling induced by LET with siRNA could be prevented by pretreatment of EGTA, chelerythrine or 17-DMAG (Fig. 4), consistent with the results showing that these inhibitors prevented LET-mediated cellular uptake of siRNA (Figs 2f and 3f, and Supplementary Fig. 6). Together with previous studies showing that intracellular actin cytoskeleton remodeling participates in cellular uptake and vesicle transport^38^, actin cytoskeleton remodeling promoted by Hsp90α and PKCγ activation would be an essential cellular event for LET-mediated cellular uptake of siRNA.

### Involvement of Rho GTPase in LET-mediated cellular uptake of siRNA

**Figure 5:**
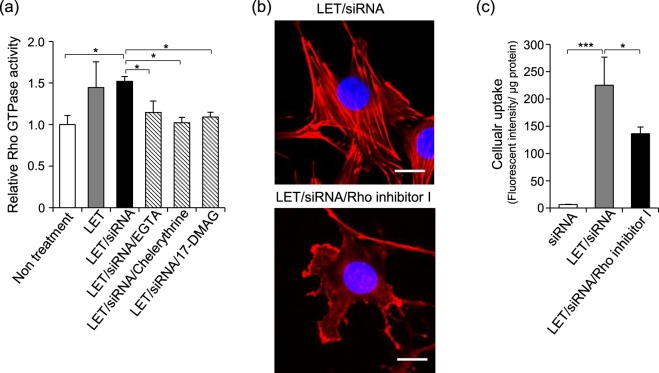
Activation of Rho GTPase by LET with siRNA. (**a**) Relative Rho GTPase activity. After 36 h of serum starvation, cells were treated with LET in presence of siRNA, and were lysed immediately after LET. Rho GTPase activity in the lysate was measured by G-Lisa activation assay (Cytoskeleton Inc.). (**b**,**c**) Cells pretreated with Rho inhibitor I for 4 h, followed by LET in presence of siRNA. (**b**) Effect of Rho inhibitor I on actin cytoskeleton remodeling induced by LET with siRNA. Red and blue signals indicate rhodamine phalloidin-labelled actin and nuclei, respectively. Scale bars indicate 20 μm. (**c**) The effect of Rho inhibitor I on LET-mediated cellular uptake of siRNA. Values represent the means of 3 individual experiments. Bars represent standard deviations. *P < 0.05 and ***P < 0.001.

Rho GTPases are a major regulator of actin remodeling^40^. To investigate whether LET activates Rho GTPase, we measured Rho GTPase activity in cells exposed to LET alone or LET with siRNA. Rho GTPase activity was significantly increased after exposure to LET with siRNA (Fig. 5a). This increased activity was prevented by pretreatment with EGTA, chelerythrine or 17-DMAG, suggesting that LET with siRNA activated Rho GTPase via Hsp90 or PKC. Moreover, pretreatment with Rho inhibitor I prevented both actin remodeling seen following LET with siRNA treatment and LET-mediated cellular uptake of siRNA (Fig. 5b,c). These results suggested that LET-mediated cellular uptake of siRNA accompanied by actin remodeling was enhanced by Rho GTPase activity promoted by Hsp90 or PKC.

## Discussion

We previously showed that LET promotes the cellular uptake of siRNA^19^. Moreover, LET has been used for the transdermal delivery of nanoparticles such as gold and poly(lactic-co-glycolic acid) (PLGA) nanoparticles^41,42^. In the present study, we examined changes in the phosphorylation status of cellular proteins to characterize the molecular mechanism underlying cellular uptake of siRNA mediated by LET. Exposure of NIH 3T3 cells to LET induced phosphorylation of various proteins that have different functions and participate in a range of biological processes (Fig. 1a,b). Among the 15 proteins that had the largest change in phosphorylation status, Hsp90α overlapped with the most cellular functions (Fig. 1c). Hsp90 functions as an ATP-dependent essential molecular chaperone in cells^43^. Post-translation modifications of Hsp90, including phosphorylation, induce structural rearrangements that promote ATP binding and subsequent hydrolysis, which in turn increase Hsp90 chaperone activity that facilitates signal transduction, protein folding and degradation, maturation of client proteins and vesicle transport^34,44^. Here we showed that the level of Hsp90α phosphorylated at Thr^5/7^ was up-regulated by LET, similar to changes induced by UV treatment (Fig. 2a,b). Thr^5/7^ of Hsp90α is located in the N-terminal domain, and phosphorylation of this region can determine the open and closed status that modulates both ATP hydrolysis and chaperone activity^43^. Although Thr^5/7^ of Hsp90α is phosphorylated by DNA activated protein kinase (DNA-PK)^44^, this kinase was not among the 139 up-regulated phosphoproteins detected by iTRAQ analysis. However, the level of phosphorylated nuclear ubiquitous casein and cyclin-dependent kinases substrate (Nucks1), which are also DNA-PK substrates^45^, in LET exposed cells was 1.525 times higher than that in non-treated cells (Fig. 1a), suggesting that Hsp90α might be phosphorylated at least in part DNA-PK. The levels of phosphorylated Hsp90α did not change in the presence of siRNA or liposomes having different physicochemical properties such as surface charge or hydration layer thickness (Fig. 2a,b), suggesting that the presence of exogenous macromolecules did not affect LET-induced Hsp90α phosphorylation. Indeed, LET promoted the cellular uptake of not only siRNA but also cationic-lipo, anionic-lipo and PEG-lipo (Fig. 2c–f). Only cationic-lipo were taken up as efficiently by untreated cells, likely due to electrostatic interactions with cellular membranes. 17-DMAG is a geldanamycin analogue that competes with ATP for Hsp90 binding, resulting in inhibition of chaperone activity and cellular events that require Hsp90 activation^46^. Pretreatment of cells with 17-DMAG prevented the cellular uptake of anionic-lipo, PEG-lipo and siRNA, suggesting that Hsp90α activity contributed to LET-mediated cellular uptake of macromolecules (Fig. 2d–f). In contrast, 17-DMAG pretreatment did not affect LET-mediated cellular uptake of cationic-lipo (Fig. 2c), indicating that Hsp90 likely does not affect electrostatic interactions of macromolecules. Although Hsp90 was previously shown to regulate intracellular vesicle transport^34^, to our knowledge this is the first report on the involvement of Hsp90 in uptake of macromolecules and more investigations are needed to characterize the role of Hsp90 in this process.

We previously reported that LET facilitates the cellular uptake of siRNA via endocytosis, and that the cellular uptake could be prevented by the non-selective cationic ion channel blocker SKF96365^19^. Here we showed that calcium influx and levels of phosphorylated PKC-γ increased in LET-exposed cells both in the absence and presence of siRNA and various liposomes (Fig. 3a,b). Thus, similar to its effect on the Hsp90 phosphorylation status, LET could also induce PKCγ phosphorylation regardless of the presence of macromolecules. The iTRAQ analysis showed that levels of phosphorylated Marcks, a PKC substrate, increased by over 2-fold in LET-exposed cells, indicating that LET induced PKC activation. Ca^2+^ chelation significantly prevented not only PKCγ phosphorylation but also cellular uptake of siRNA (Fig. 3d–f), suggesting that PKC activation following calcium influx contributes to LET-mediated cellular uptake of siRNA.

A study by Lu X. *et al*. proposed a synergistic interaction between PKCγ and Hsp90α wherein Hsp90α forms a chaperone complex with PKCγ that in turn promotes its translocation from the cytosol to the plasma membrane and subsequent activation^47^. At the membrane, Hsp90α Thr^115^, Thr^425^ and Thr^603^ residues are specifically phosphorylated by PKCγ, followed by a decrease in Hsp90 chaperone activity upon dissociation of PKCγ from Hsp90 in the cytoplasm. However, we showed that LET-mediated cellular uptake of siRNA could be prevented by pretreatment with 17-DMAG (Fig. 2b,f), suggesting that phosphorylated Hsp90α chaperone activity was present during PKCγ activation. Moreover, pretreatment with EGTA or the PKC inhibitor chelerythrine did not inhibit Hsp90α Thr^5/7^ phosphorylation induced by LET with siRNA (Supplementary Fig. S4a,b). Although further investigations are needed, PKCγ might not be the upstream signaling molecule for Thr^5/7^ phosphorylation of Hsp90α in LET-mediated cellular uptake of siRNA and PKCγ and Hsp90α would differentially facilitate LET-mediated cellular uptake of siRNA.

**Figure 6:**
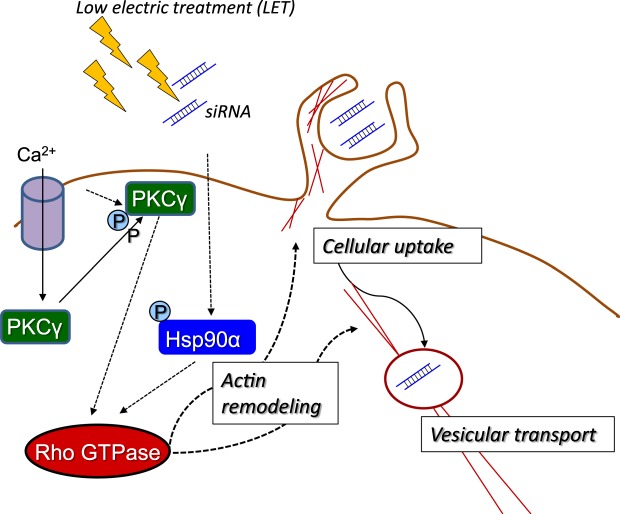
Mechanism and signaling pathway for LET-mediated cellular uptake of siRNA. LET with siRNA induces the phosphorylation of Hsp90α and PKCγ, followed by the activation of Rho GTPase. The action remodeling induced by Rho GTPase promotes the cellular uptake and vesicular transport of siRNA.

We previously reported that LET-mediated cellular uptake of siRNA could be prevented by the macropinocytosis inhibitor amiloride^19^. Macropinocytosis, which is non-selective fluid phase endocytosis, is important for cellular entry of amino acids, virus and electro-transfected plasmid DNA, and is accompanied by actin cytoskeleton remodeling^48–50^. We showed that actin cytoskeleton remodeling was induced by LET with siRNA, and this remodeling could be inhibited by pretreatment with EGTA, chelerythrine or 17-DMAG (Fig. 4). Together with our findings that LET-mediated cellular uptake of siRNA was prevented by pretreatment with EGTA or 17-DMAG (Figs 2f and 3f), these results suggested that PKC activation following Ca^2+^ influx in the presence of Hsp90α induced actin remodeling, leading to the promotion of cellular uptake of siRNA mediated by LET. Additionally, Rho GTPase regulation of actin remodeling was activated by LET with siRNA, which was also inhibited by pretreatment with EGTA, chelerythrine or 17-DMAG (Fig. 5a). Pretreatment with the Rho inhibitor I prevented not only actin remodeling, but also cellular uptake of siRNA mediated by LET (Fig. 5b,c). Rho GTPase is a subfamily of small GTP-binding proteins of the Ras superfamily, and family members Cdc42, Rac1 and RhoA regulate membrane trafficking through the formation of filopodia, lamellipodia and stress fibers, respectively^40,51^. In the initial phase of macropinocytosis, extensive actin remodeling is required to form the dorsal membrane and peripheral ruffles^40^. Rho GTPase is also involved in formation of ruffles by binding and activating actin nucleators^40^. Therefore, activation of Rho GTPase is vital for the promotion of macropinocytosis and membrane trafficking. Hsp90 was shown to activate Rho GTPase indirectly by protecting functional activators from proteasomal degradation^52^. Moreover, PKCγ activates Rho GTPase thorough phosphorylation of guanine nucleotide exchange factor (GEF) that favors the GTP-bound over GDP-bound form^53^. Taken together, these findings show that Hsp90α and PKCγ promote actin remodeling via the activation of Rho GTPase (Fig. 6).

In conclusion, in this study we demonstrated that LET with siRNA induced the phosphorylation of Hsp90α and PKCγ, which would be both involved in activation of Rho GTPase, indicating that actin remodeling would be important for LET-mediated cellular uptake of siRNA.

## Materials and Methods

### Materials

1,2-dioleoyl-3-trimethylammonium-propane (DOTAP) and 1,2-dioleoyl-sn-glycero-3-phosphoethanolamine-N (lissamine rhodamine B sulfonyl) (Rh-PE) were purchased from Avanti Polar Lipids (Alabaster, AL, USA). Egg phosphatidylcholine (EPC) and N-(carbonyl-methoxypolyethylene glycol 2000)-1,2-distearoyl-sn-glycero-3-phosphoethanolamine (PEG-DSPE) were obtained from NOF Corporation (Tokyo, Japan). Cholesterol, dihexadecyl phosphate (DCP) and PKH26 were purchased from Sigma-Aldrich (St. Louis, MO, USA). Anti-luciferase siRNA (21-mer, 5′-GCGCUGCUGGUGCCAACCCTT-3′, 5′-GGGUUGGCACCAGCAGCGCTT-3′: anti-Luc) and Rhodamine labelled anti-GFP siRNA (21-mer, 5′-GCUGACCCUGAAGUUCAUCTT-3′, 5′-GAUGAACUUCAGGGUCAGCTT-3′: anti-GFP) were obtained from Invitrogen Life Technologies (Carlsbad, CA, USA). Fluo-4 calcium kits were obtained from Dojindo Molecular Technologies, Inc. (Rockville, MD, USA). Rhodamine phalloidin, G-Lisa activation assay kit and Rho inhibitor (C3 transferase) were purchased from Cytoskeleton, Inc. (Denver, CO, USA). Alvespimycin HCl (17-DMAG) was obtained from BSP Bioscience, Inc. (San Diego, USA). Chelerythrine chloride was purchased from Wako Pure Chemical Industries, Ltd. (Osaka, Japan). 5′-O-[(4-Cyanophenyl)methyl]-8-[[(3,4-dichlorophenyl)methyl]amino]-adenosine (VER-155008), and Gö6983 were purchased from Sigma-Aldrich (St. Louis, MO, USA). Phospho HSP90α Thr^5/7^ antibody, phospho PKC gamma (PKCγ) Thr^514^ antibody and β-Actin antibody were purchased from Cell Signaling Technology (Danvers, MA, USA).

### Cell culture

Mouse fibroblast NIH 3T3 cells were obtained from RIKEN BRC Cell Bank (Wako, Japan). The cells were cultivated in Dulbecco’s modified Eagle’s medium (DMEM) supplemented with 10% fetal bovine serum (FBS) at 37 °C, 21% O_2_, and 5% CO_2_ under humidified conditions.

### Preparation of liposomes

Liposomes were prepared by a simple lipid film hydration method according to our previous report^22^. Briefly, for preparation of cationic liposomes, EPC and DOTAP dissolved in ethanol were mixed at a molar ratio of 7.6:1, and the ethanol was evaporated under a nitrogen stream to form a thin lipid film. PBS was added to the dried lipid film (total lipid concentration 10 mM) and the suspension was sonicated using a bath-type sonicator (ULTRASONIK 14B, NEY, CA). The anionic liposomes and PEG liposomes were prepared as described above and contained the following lipid compositions: EPC/DCP (9/1 mol/mol) and EPC/Cholesterol/PEG-DSPE (1.85/1/0.15 mol/mol/mol). Liposome particle sizes and ζ-potentials were examined by dynamic light scattering and laser Doppler, respectively, using a Zetasizer nano (Malvern Ins., Ltd.), and are summarized in Supplementary Table S2.

### Low electric treatment (LET) of cells

The number of cells used in each experiment is described below. After 18 h of cultivation, cells were washed with PBS, and 1 ml serum-free DMEM containing the indicated macromolecules (e.g., cationic liposomes, anionic liposomes or PEG liposomes; final lipid concentration: 25 μM) or anti-luciferase siRNA (100 pmol) were added to the cells. Ag-AgCl electrodes with a 2.5 cm^2^ surface area (3 M Health Care, Minneapolis, MN, USA) were placed into the dish and cells were treated with a constant current of 0.34 mA cm^−2^ for 15 min.

### iTRAQ, Gene Ontology (GO) slim and STRING analysis

For iTRAQ analysis, NIH 3T3 cells were cultivated at 1.5 × 10^5^ cells per dish on 35 mm dishes for 24 h. After washing with PBS (−), cells in PBS (−) were exposed to LET at a constant current of 0.45 mA cm^−2^ for 15 min. After washing with PBS (−), cells located under the electrode of the cathode were collected and frozen under liquid nitrogen. Untreated cells and cells exposed to LET from three independent experiments were pooled into treated and untreated samples. iTRAQ analysis was carried out at Filgen, Inc. (Nagoya, Japan) according to the manufacturer’s protocol accompanying the iTRAQ kit (AB SCIEX, Tokyo, Japan). Briefly, proteins extracted from each of the pooled samples were reductively alkylated and digested with trypsin. The resulting peptide samples were demineralized and labeled with iTRAQ. In this analysis, samples from LET-exposed and untreated cells were labeled with a 116 isobaric tag and 114 isobaric tag, respectively. After concentrating the phosphorylated peptides, identification and relative quantification of phosphorylated proteins were carried out by AB SCIEX Triple TOF^TM^ 5600 System with ProteinPilot^TM^ software 4.1 (AB SCIEX). The thresholds in this study were set as described below. Up-regulated or down-regulated phosphorylated proteins in LET samples were defined as >1.2-fold change relative to untreated cells and a Unused Prot Score >0.62 to show reliabilities of identified proteins. To understand the biological processes, molecular functions and cellular components associated with the phosphorylated proteins that were up-regulated by LET, a GO analysis was carried out at Filgen, Inc. (Nagoya, Japan) using the MicroArray Data Analysis Tool Ver 3.2 (Filgen, Inc). GO slim analysis is a useful tool for understanding the functional role of changed proteins group by statistically addressing the frequency of GO term-attached proteins^54^. To determine interactions among the 15 proteins showing the largest upregulation in phosphorylation following LET, STRING analysis was performed by using STRING ver 10.5 (ELIXIR Core Data Resources).

### Western blotting

Western blotting was performed according to our previous report^22^. Briefly, 4 × 10^5^ NIH 3T3 cells were seeded in 35 mm dishes. After 18 h of cultivation, the cells were washed with PBS and 1 ml serum-free DMEM containing liposomes (final lipid concentration: 25 μM) or anti-luciferase siRNA (100 pmol) was added before LET with a constant current of 0.34 mA cm^−2^ for 15 min. Immediately after LET, the cells were washed with PBS and treated with lysis buffer (25 mM Tris-HCl [pH 6.5], 1% [v/v] glycerol, 1% [v/v] SDS, 5% 2-mercaptoethanol and the phosphatase inhibitor cocktail PhosSTOP [Sigma Aldrich]). The amount of protein in the samples was determined by BSA Protein Assay (Thermo Fisher Scientific Inc., Waltham, MA, USA) and equal amounts of proteins (40 μg/lane) were loaded on 10% SDS-PAGE gels. Proteins were separated by SDS-PAGE and transferred to polyvinylidene difluoride (PVDF) membranes that were then blocked by 2% BSA in Tween solution (500 mM Pi buffer, 150 mM NaCl, 0.16% [v/v] Tween 20). Membranes were incubated with primary antibody (diluted and incubated according to the manufacturer’s instructions) followed by incubation with the indicated secondary antibody diluted 1:2000. The blots were detected using ECL Western blotting detection reagent (GE Healthcare, Waukesha, WI) and a C-DiGit blot scanner (LI-COR Biosciences GmbH, Germany). Quantitative analysis of blots was performed by Image Studio Digits software (ver. 5) (LI-COR Biosciences GmbH, Germany). In this analysis, β-actin was used as an internal control.

### Treatment with pharmacological inhibitors and analysis of cellular uptake of macromolecules

NIH 3T3 cells (1 × 10^5^) were seeded in 35 mm dishes. After 18 h of cultivation, cells were washed with PBS and then 1 ml serum free DMEM containing either EGTA (5 mM), 17-DMAG (10 μM), chelerythrine (2 μM) or Rho inhibitor I (2 μg/ml) was added into the dish, which was then incubated for 30 min, 1 h, 1 h or 4 h. After incubation, liposomes (final lipid concentration; 25 μM, ratio of rhodamine-PE labelled liposomes to unlabeled liposomes: 1:99) or rhodamine-labelled siRNA (100 pmol) was added to the dishes and the cells were exposed to LET with a constant current of 0.34 mA cm^−2^ for 15 min. After LET, cells were incubated at 37 °C for 45 min before washing with PBS and lysing with reporter lysis buffer (Promega) according to the manufacturer’s protocols. The fluorescence intensity of the cell lysate was measured using a microplate reader Infinite (Tecan Group Ltd., Mannedorf, Switzerland) at excitation and emission wavelengths of 546 nm and 590 nm, respectively.

### Evaluation of intracellular Ca^2+^ following LET using fluo-4 fluorescent dye

The intracellular amount of Ca^2+^ was evaluated using fluo-4 fluorescent dye according to the manufacturer’s protocol. Briefly, NIH 3T3 cells (5 × 10^4^ cells) were seeded in 35 mm glass bottom dishes coated with 0.002% poly-L-lysine (PLL). After 18 h of cultivation, cells were washed with PBS before 1 ml loading buffer was added to the dish, which was incubated at 37 °C for 1 h. For EGTA treatment, cells were pre-treated with 5 mM EGTA for 30 min. After removing loading buffer, 1 ml pre-warmed recording medium containing liposomes (final lipid concentration: 25 μM) or anti-luciferase siRNA (100 pmol) was added to the dish, followed by LET (0.34 mA/cm^2^) for 2 min. Immediately after LET, the fluorescence signals corresponding to the amount of intracellular Ca^2+^ was observed by Confocal Laser Scanning Microscopy (CLSM) A1R + (Nikon Co., Ltd., Japan).

### Actin staining of cells after LET with siRNA

For actin staining, NIH 3T3 cells (5 × 10^4^) were seeded in 35 mm glass bottom dishes coated with 0.002% PLL. After 18 h of cultivation, cells were washed with PBS and 1 ml serum free DMEM containing liposomes (final lipid concentration 25 μM) or anti-luciferase siRNA (100 pmol) was added before the LET with a constant current of 0.34 mA cm^−2^ for 15 min. Immediately after LET, cells were washed with PBS and fixed with 4% paraformaldehyde for 10 min at room temperature. After washing with PBS, cells were permeabilized with 1% Triton X-100 at room temperature for 10 min. The cells were then incubated with rhodamine phalloidin at a final concentration of 100 nM for 30 min in dark. After washing, cells were mounted in VECTASHIELD with DAPI and observed by CLSM A1R+ (Nikon Co., Ltd., Japan).

### Measurement of Rho GTPase activity

NIH 3T3 cells (4 × 10^5^ cells) were seeded on 35 mm dishes. After 36 h of serum starvation for reducing high basal level of Rho GTPase activity of cells, the cells were washed with PBS and 1 ml serum-free DMEM containing 100 pmol anti-luciferase siRNA. For inhibitory treatment, cells were pretreated with EGTA (5 mM, 30 min), chelerythrine (2 μM, 1 h), or 17-DMAG (10 μM, 1 h) before addition of siRNA. After LET, the cells were lysed and the Rho GTPase activity in the whole cell lysate was measured using a G-Lisa activation assay (Cytoskeleton Inc.) according to the manufacturer’s instructions.

### Statistical analysis

Statistical analysis was conducted using one-way ANOVA followed by Turkey-Kramer HSD test. *P* values < 0.05 were considered to be significant.

## Supplementary information

Supplementary Information
