# Supplementary material for: Low Electric Treatment activates Rho GTPase via Heat Shock Protein 90 and Protein Kinase C for Intracellular Delivery of siRNA
Source: Sci Rep. 2019 Mar 11;9:4114. doi: 10.1038/s41598-019-40904-z (PMC6412017; doi:10.1038/s41598-019-40904-z)
Supplement: Supplementary file 1 — Supplementary Information [file 41598_2019_40904_MOESM1_ESM.pdf]

Supplementary Information

**Title:** Low Electric Treatment activates Rho GTPase via Heat Shock Protein 90 and Protein Kinase C for Intracellular Delivery of siRNA

Author names:
Mahadi Hasan[1,2,3], Susumu Hama[1] and Kentaro Kogure[2]

Affiliations:
[1]Department of Biophysical Chemistry, Kyoto Pharmaceutical University, Kyoto 607-8414, Japan;
[2]Department of Pharmaceutical Health Chemistry, Tokushima University Graduate School of Biomedical Sciences, Tokushima 770-8505 Japan .
[3]JSPS International Research Fellow

Supplementary Table. S1

**GO slim analysis of GO term-attached proteins among the top Top 15 phosphorylated proteins upregulated by LET**

| Names | Cellular component | Molecular function | Biological process |
|---|---|---|---|
| Hnrnpc | Cell; intracellular; organelle; cytoplasm; nucleus; cytosol; nucleoplasm; protein complex | RNA binding; mRNA binding | N.D |
| Pdap1 | Extracellular space, extracellular region | RNA binding | N.D |
| Mtdh | Cell; intracellular; organelle; cytoplasm; nucleus; endoplasmic reticulum; nucleolus | RNA binding | N.D |
| Hmgb1 | Cell; intracellular; organelle; cytoplasm; nucleus; plasma membrane; extracellular space; extracellular region | Lyase activity | N.D |
| Marcks | Cell; intracellular; cytoplasm | N.D | N.D |
| Eef1b2 | Cell; intracellular; organelle; cytoplasm; endoplasmic reticulum; | N.D | N.D |
| Hsp90aa1 | Cell; intracellular; organelle; cytoplasm; nucleus; cytosol; protein complex | RNA binding; unfolded protein binding; ATPase activity | N.D |
| Huwe1 | Cell; intracellular; organelle; cytoplasm; nucleus; cytosol; nucleoplasm; | RNA binding; DNA binding | Cell differentiation |

N.D.: Not determined

Supplementary Table. S2

**Physicochemical properties of liposomes**

| | Particle size (nm) | ζ-potential (mV) |
|---|---|---|
| Cationic lipo | 96±8.1 | 10±1.9 |
| Anionic lipo | 97±14 | -17±3.1 |
| PEG-lipo | 87±7.8 | -1.8±0.47 |

Supplementary Fig. S1

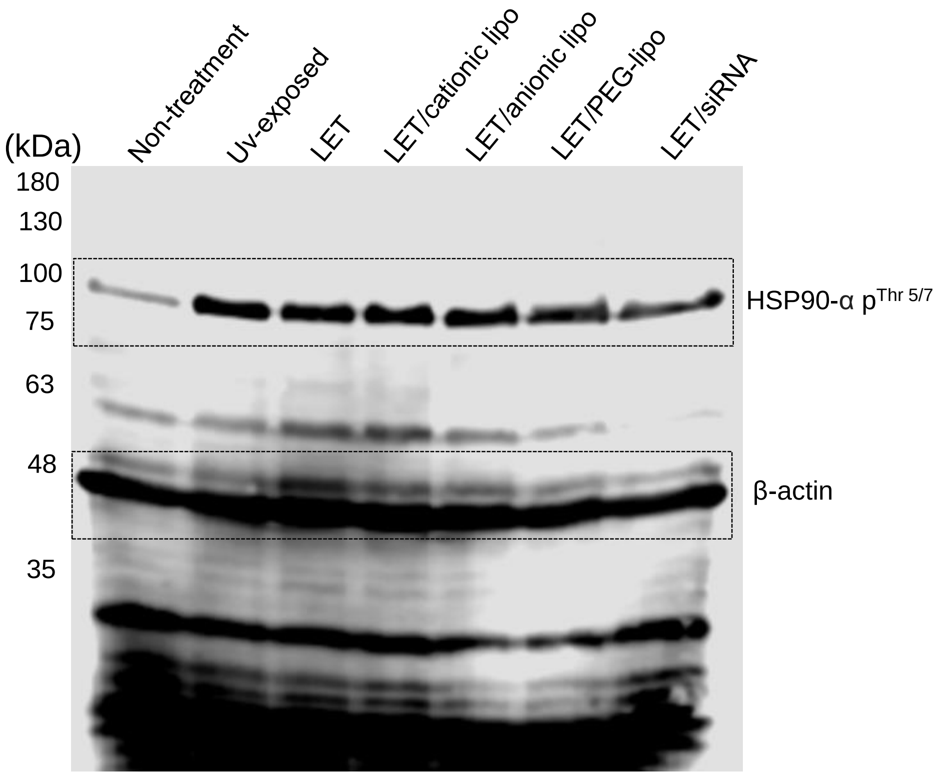


**Full-length blots regarding figure 2 (a)**

Supplementary Fig. S2

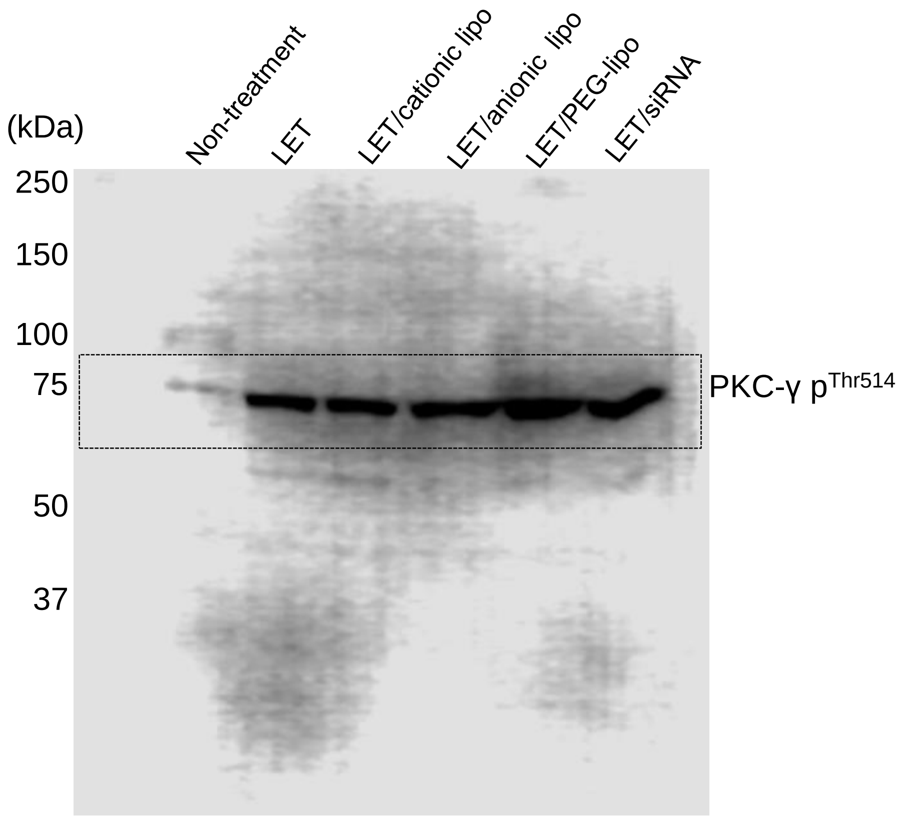


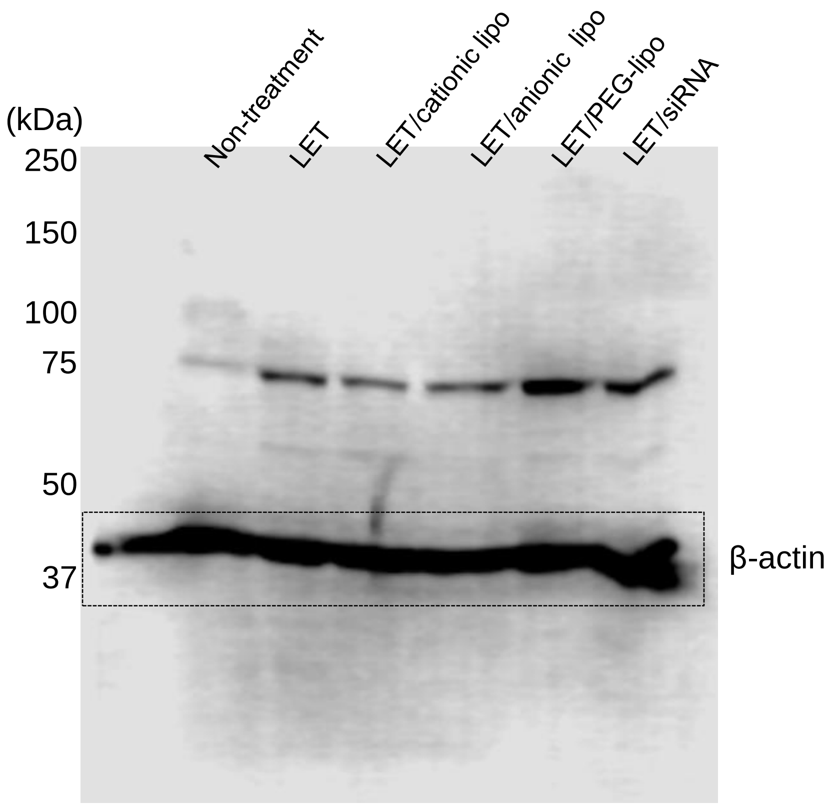


**Full-length blots regarding figure 3 (a)**

Supplementary Fig. S3

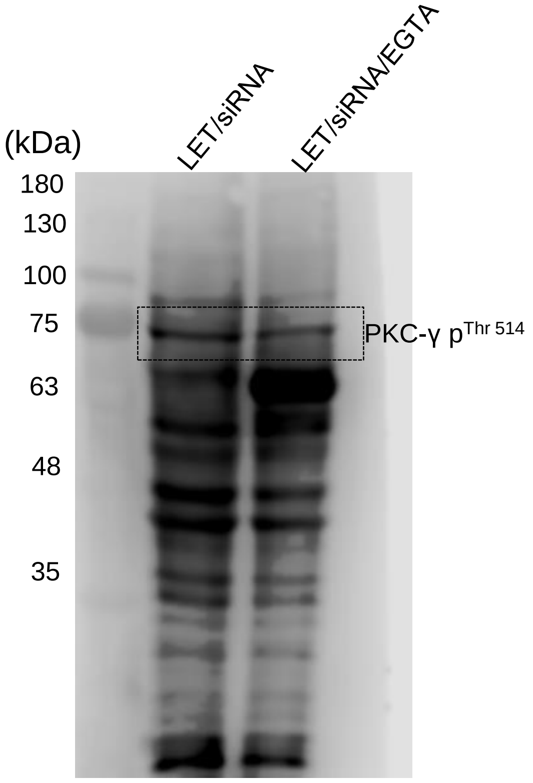


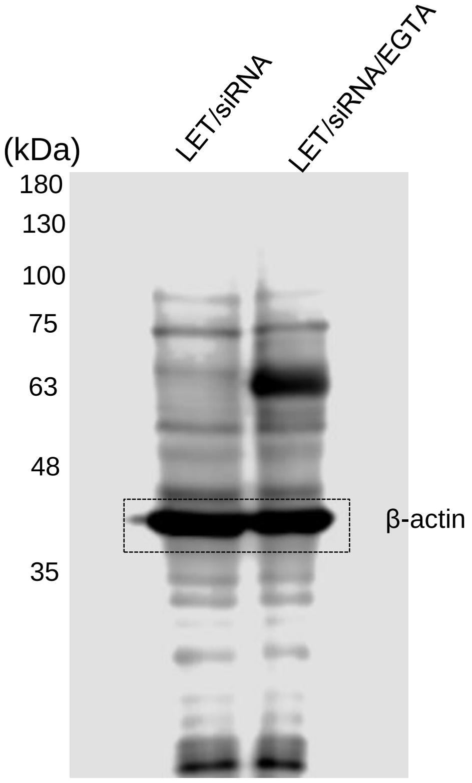


**Full-length blots regarding figure 3 (d)**

Supplementary Fig. S4

a

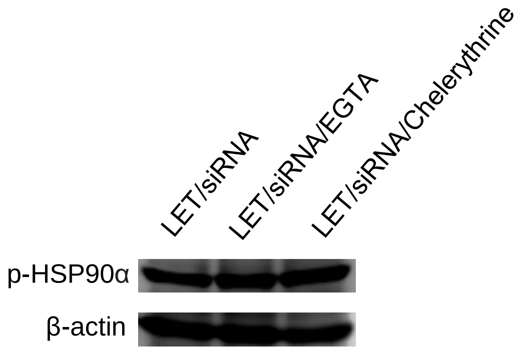


b

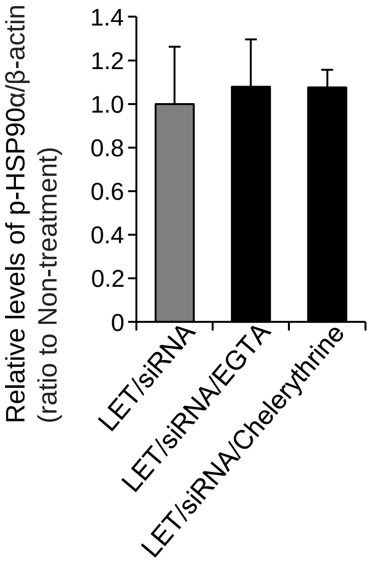


c

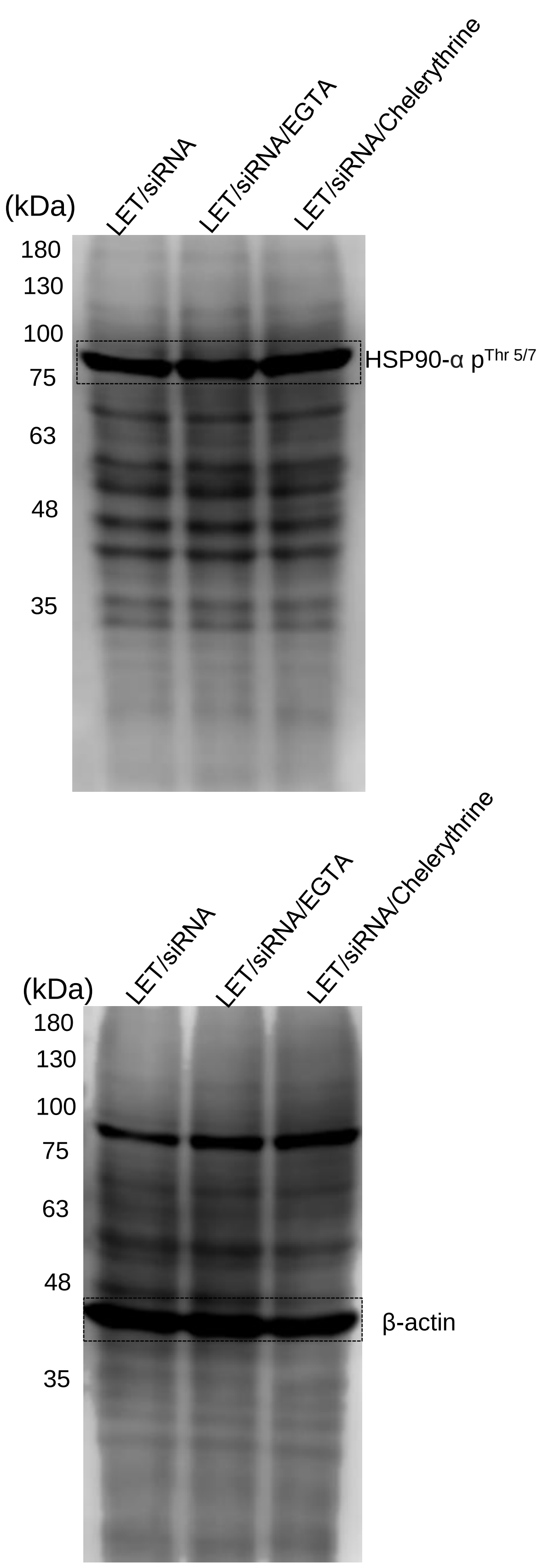


**Effects of EGTA and chelerythrine on Hsp90α phosphorylation induced by LET with siRNA**

Cells were pretreated with EGTA or chelerythrine for 30 min prior to the LET in presence of siRNA. Phospho-Hsp90α (Thr5/7) levels were determined by western blotting. β-actin was used as a loading control.(a) Typical images from at least 3 individual experiments.(b) Relative levels p-Hsp90α/β-actin. Data are shown as the ratio to non-treatment. Values represent the means of 3 individual experiments. Bars represent standard deviations. (c) Full-length blots regarding Fig. S4a

Supplementary Fig. S5

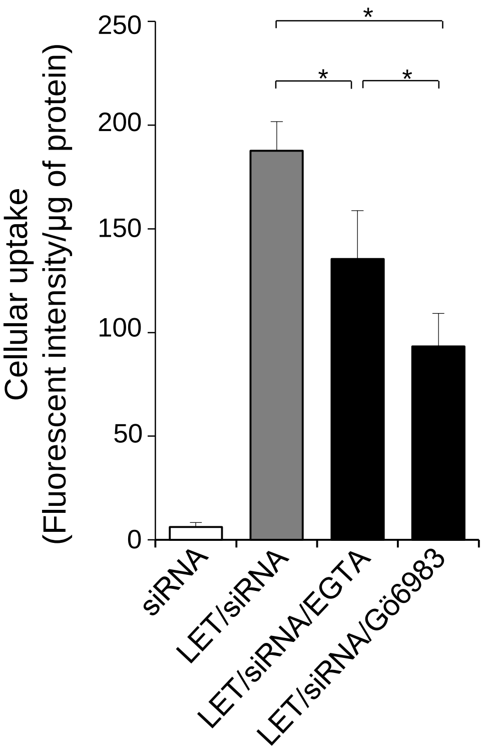


**Effects of EGTA or PKC inhibitor Gö6983 on cellular uptake of siRNA**

NIH3T3 cells were pretreated with EGTA or PKC inhibitor Gö6983 prior to LET in presence of rhodamine-labeled siRNA. After incubation for 45 min, cells were lysed, and fluorescence intensity in the lysate was measured. Values represent the means of 3 individual experiments. Bars represent standard deviations. *$p$ < 0.05.

Supplementary Fig. S6

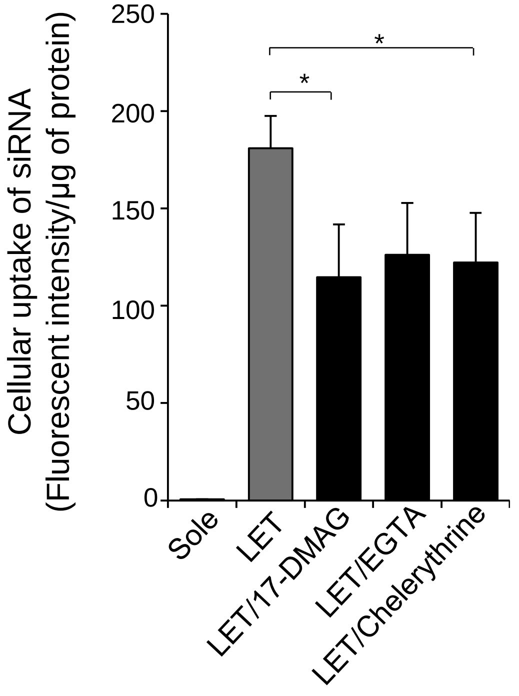


**Effects of Hsp90 inhibitor, EGTA or PKC inhibitor on cellular uptake of siRNA in B16-F1 cells**

B16-F1 cells were pretreated with 17-DMAG, EGTA or Chelerythrine prior to LET in presence of rhodamine-labeled siRNA. After incubation for 45 min, cells were lysed, and fluorescence intensity in the lysate was measured. Values represent the means of 3 individual experiments. Bars represent standard deviations. $^*p < 0.05$.
